# HS-Associated *Pasteurella multocida* Infection Disrupts Gut Microbiota and Metabolism in Mice

**DOI:** 10.3390/microorganisms14010066

**Published:** 2025-12-28

**Authors:** Kewei Li, Chao Jin, Haofang Yuan, Muhammad Farhan Rahim, Xire Luosong, Tianwu An, Jiakui Li

**Affiliations:** 1 College of Veterinary Medicine, Huazhong Agricultural University, Wuhan 430070, China; lizangxi97@webmail.hzau.edu.cn (K.L.); 2022302120148@webmail.hzau.edu.cn (C.J.); yhf@webmail.hzau.edu.cn (H.Y.); farhan092@webmail.hzau.edu.cn (M.F.R.); 2 Xizang Veterinary Biological Pharmaceutical Factory, Lhasa 850000, China; m13908940656@163.com; 3 Sichuan Academy of Grassland Sciences, Chengdu 611731, China; 4 Jiangxia Laboratory, Wuhan 430200, China; 5 College of Animal Science and Technology, Xizang Agricultural and Animal Husbandry University, Nyingchi 860000, China

**Keywords:** hemorrhagic septicemia, *Pasteurella multocida*, gut microbiota, metabolism, tyrosine, dopamine

## Abstract

*Pasteurella multocida* serotype B:2 is a primary agent of hemorrhagic septicemia (HS) in livestock, and the strain NQ01 isolated from yaks highlights its cross-species impact. In this study, a murine intranasal infection model was established using *P. multocida* NQ01 to assess how acute respiratory infection perturbs gut homeostasis. Mice were intranasally inoculated with NQ01, and at 36 h post-infection, ileal tissues and cecal contents were collected for histopathological examination, 16S rRNA gene sequencing, and untargeted metabolomic analysis. Histopathology revealed obvious acute bronchopneumonia but no overt ileal damage. However, 16S rRNA sequencing of cecal microbiota showed significant dysbiosis: microbial diversity was reduced and community composition shifted, including decreased short-chain fatty-acid-producing taxa and increased opportunistic genera. Metabolomic profiling detected 1444 significantly altered cecal metabolites, and pathway analysis indicated marked disruption of amino acid metabolism, notably the tyrosine metabolism pathway. Key tyrosine pathway metabolites were dysregulated (e.g., elevated L-tyrosine and dopamine with reduced L-DOPA), indicating a breakdown of this metabolic pathway. These findings demonstrate that acute respiratory *P. multocida* infection profoundly disturbs gut microbiota and metabolism, underscoring disruption of the gut–lung axis. This study provides new insight into the systemic consequences of HS-associated *P. multocida* infection and offers a basis for exploring the gut–lung interaction in hemorrhagic septicemia pathogenesis.

## 1. Introduction

*Pasteurella multocida* (*P. multocida*) is an important opportunistic pathogen that resides in the upper respiratory tract of various hosts, including poultry, livestock, wild animals, and humans [1]. Traditionally *P. multocida* strains are classified into five capsular serogroups (A, B, D, E, and F) and sixteen lipopolysaccharide (LPS) serotypes (1–16), with further subdivision into eight genotypes (L1–L8) based on unique LPS outer core biosynthesis loci [2,3,4]. Bovine-associated *P. multocida* are related to two diseases: bovine respiratory disease (BRD) and hemorrhagic septicemia (HS). BRD is caused by serotype A and D strains and characterized as a respiratory disease that suddenly occurs in a high-stress environment, such as transportation and co-mingling [5]. HS is mainly caused by two specific serotypes, B and E, whose outbreaks among cattle and buffalo populations have resulted in significant losses in Asia and Africa [6,7]. Previous observations showed that HS presents with an acute or peracute clinical course, with death occurring within a few hours to days after the onset of clinical signs [8,9]. Typical symptoms include persistent high fever, dyspnea, nasal discharge, and lacrimation, accompanied by subcutaneous edema in the submandibular regions. Pathological examination reveals generalized hemorrhages in multiple organs with evident coagulopathy [7]. *P. multocida* is a commensal organism that resides in the upper respiratory tract, belonging to the airway microbiota. Translocation and proliferation are considered important initial pathogenic processes for *P. multocida,* and the infectious dose is regarded as the decisive factor for the onset of HS [8]. When the host suffers from environmental stress, mucosal damage, or infection by any other pathogen, the airway microbiota can be disrupted. Consequently, *P. multocida* proliferates in the soft tissues or may be phagocytosed by macrophages and transported to the lymph nodes, where they multiply and disseminate to surrounding tissues [10].

Although airway microbiota play a crucial role in host health and disease, the research in this field has lagged significantly behind that of the gut microbiota. This disparity stems largely from the limitations of in vivo sampling, the constraints of culture-based techniques, and potential interference from upper respiratory tract contamination [11]. Although the gastrointestinal tract is relatively independent of the respiratory tract, they are interconnected as a shared mucosal immune system through the gut–lung axis [12]. The gut microbiota has been proven to play a positive role in the establishment of pulmonary immunity, which protects against pathogenic infection [13,14,15]. Gut microbiota dysbiosis has been proven to be related to the initial onset and continuous aggravation of chronic pulmonary diseases, such as chronic obstructive pulmonary disease (COPD) [16], asthma [17], bronchiectasis [18], cystic fibrosis pulmonary exacerbations [19], etc. The gut microbiota responds more rapidly and exerts a more direct impact on acute pulmonary disease. This is evidenced by the immediate translocation of gut bacteria to the lungs in experimental sepsis mice and patients with established acute respiratory distress syndrome (ARDS) [20]. The metabolites produced by the gut microbiota are crucial to balance the host–microbiota homeostasis. As widely reported, short-chain fatty acids (SCFAs) synthesized by the gut microbiota are recognized as the protective metabolites against respiratory pathogens infection [21,22]. Additionally, the gut metabolites corresponding to lipid metabolism, purine metabolism, and the pentose phosphate pathway are proven to have a significant correlation with pulmonary diseases [23,24].

In this study, we aim to explore the alterations of gut microbiota and metabolites following intranasal infection with NQ01, which is a *P. multocida* serotype B:2 strain isolated from yak. Through 16S rRNA gene sequencing and untargeted liquid chromatography–tandem mass spectrometry, we reveal the dynamic distribution of bacterial communities and metabolites in the cecum of mice. Overall, our findings provide a new insight into the systemic effects of HS-associated *P. multocida* infection and a theoretical reference regarding the pathogenesis of this type of bacterial infection.

## 2. Materials and Methods

### 2.1. Ethics Statement

All animal experiments carried out in the research adhere to the guidance of the animal ethical committee of Huazhong Agricultural University (Permission Number: HZAUMO-2025-0129).

### 2.2. Cultivation and Maintenance of Bacterial Strains

The *P. multocida* strain used in this study was isolated from yaks named NQ01 (NCBI accession No. GCA_048814815.1). NQ01 is maintained in the Clinical Veterinary Internal Medicine Laboratory at Huazhong Agricultural University. A lyophilized strain was reconstituted with 1 mL of tryptic soy broth (TSB) and with 5% bovine serum; after that, it was inoculated onto TSA with 5% defibrinated sheep blood and incubated at 37 °C for 24 h. A pure single colony was identified and amplified for serial passage under the same conditions. Once the bacterial cultures reached the stationary growth phase, tenfold serial dilutions were prepared using phosphate-buffered saline (PBS).

### 2.3. Animal Experiment and Sample Collection

A total of 12 healthy 6-week-old female BALB/c mice were randomly divided into the CT group and the Pm group (sample size *n* = 6 per group), which were raised in the laboratory animal center of Huazhong Agricultural University. During the experimental period, mice were housed under standard conditions, including a recommended temperature range of 22–26 °C, relative humidity of 50–60%, and a 12-h light/dark cycle. Animals had free access to standard chow and water ad libitum. Following a 5-day acclimatization period, mice in the *Pm* group were anesthetized and intranasally inoculated with twice the LD_50_ dose of *NQ01* (approximately 3.82 × 10^5^ CFU per mouse) in a volume of 20 μL. Control (*CT*) group mice received an equal volume of phosphate-buffered saline (PBS) via the same route. The LD_50_ value (9.53 × 10^6^ CFU/mL) was previously determined in our earlier study [25]. By 36 h post-infection (hpi), mice in the Pm group exhibited signs of lethargy, ruffled fur, and rapid breathing, in contrast to the normal behavior observed in the control group. Both CT and Pm mice were humanely euthanized by rapid cervical dislocation and cecal content samples were collected for subsequent gut microbiota sequencing and metabolomic profiling. Simultaneously, the lung and a small segment of the ileum were excised and stored at −80 °C for subsequent assessment of organ structural damage.

### 2.4. Histological Observations

Fresh mouse tissues including lungs and ileums from each group (*n* = 6) were fixed into 4% paraformaldehyde at room temperature for over 48 h. After being dehydrated and cleansed in ethanol and xylene, the fixed tissues were embedded in paraffin. Histological sections were prepared and stained with hematoxylin and eosin (H&E). Tissue sections were observed by inverted microscope to evaluate histopathological changes.

### 2.5. 16S rRNA Sequencing and Bioinformatics Analysis

The acquired cecal contents were unfrozen, and the microbiota DNA extraction was performed under aseptic conditions strictly following the manufacturer’s protocol (TIANGEN, Beijing, China). The concentration and integrity of the DNA were assessed using a micro-spectrophotometer (GeneCompany, Beijing, China) and 1% agarose gel electrophoresis. The V3/V4 hypervariable region of the bacterial DNA were amplified with primers (338F: ACTCCTACGGGAGGCAGCA; 806R:GGACTACHVGGGTWTCTAAT). The PCR products were verified by 1.8% agarose gel electrophoresis. Target amplicons, identified as bright bands within the 400–500 bp range, were excised and purified with a Gel Extraction Kit (Monarch, Ipswich, MA, USA). The purified PCR products were then utilized to generate sequencing DNA libraries. The constructed sequencing libraries were quality-controlled using the Qsep 400 system (BiOptic, Hsinchu City, Taiwan, China). Libraries meeting the quality standards were sequenced on the Illumina NovaSeq 6000 platform (Illumina, San Diego, CA, USA).

The raw data obtained from sequencing were initially processed using Trimmomatic (v0.33) for quality control. Primer sequences were then identified and removed with Cutadapt v1.9.1 to generate clean reads. Denoising was performed using the DADA2 algorithm within QIIME2 v2020.6, including paired-end sequence merging and chimera removal, to obtain high-quality non-chimeric reads. These refined sequences were subsequently clustered into amplicon sequence variants (ASVs) for downstream analyses, including diversity assessment, differential abundance analysis, correlation analysis, and functional prediction. Alpha diversity analysis was conducted using QIIME to evaluate variations in microbial richness and diversity within the gut microbiota across samples, including calculations of Chao1, Ace, Shannon, and Simpson index. Beta analysis, including principal component analysis (PCA), principal coordinates analysis (PCoA) and non-metric multidimensional scaling (NMDS), was applied to explore the differences in the composition of the gut microbiota. Matastats analysis was employed to discern the differential bacteria between groups by software.

### 2.6. Samples Preparation and Metabolomics Analysis

The sample Cecal contents (50 mg per sample) were collected and mixed with 1000 μL of the extraction solution (methanol/acetonitrile/H_2_O = 2:2:1) containing the internal standard (with a ratio of 1000:2). Then, they were processed for 10 min using a 45 Hz grinder with ceramic beads. After being left at −20 °C for one hour, the samples were centrifuged for 15 min (4 °C, 12,000 rpm). The supernatant was dried in a vacuum concentrator and then resolubilized using 160 μL of the extract solution (acetonitrile/H_2_O = 1:1). Then, the extract was subjected to ultrasound treatment for ten minutes and then centrifuged again. The supernatant was collected for testing.

The liquid chromatography–tandem mass spectrometry (LC-MS/MS) contained a high-performance liquid chromatograph (Acquity I-Class PLUS, Waters, Milford, CN, USA) and a high-resolution mass spectrometer (Xevo G2-XS QTof, Waters). The raw data collected by MassLynx V4.2 were processed through Progenesis QI software (V3.0) for tasks such as peak extraction and peak alignment. Based on the METLIN database (https://metlin.scripps.edu/, accessed on 11 November 2025), substance identification was performed using Progenesis QI software. PCA and orthogonal projections to latent structures discriminant analysis (OPLS-DA) were performed to investigate the variation in metabolism among different groups. The variable importance in projection (VIP) > 1 or *p* < 0.05 were used to screen the metabolites with significant difference.

### 2.7. Statistical Analysis

Statistical data figures were created using GraphPad Prism 9.0 software. Student’s *t*-test and analysis of variance (ANOVA) were employed for data with the SPSS software (version 23.0). *p*-value < 0.05 was set as statistical significance.

## 3. Result

### 3.1. NQ01 Infection Induces No Significant Changes in Murine Ileal Morphology

**Figure 1:**
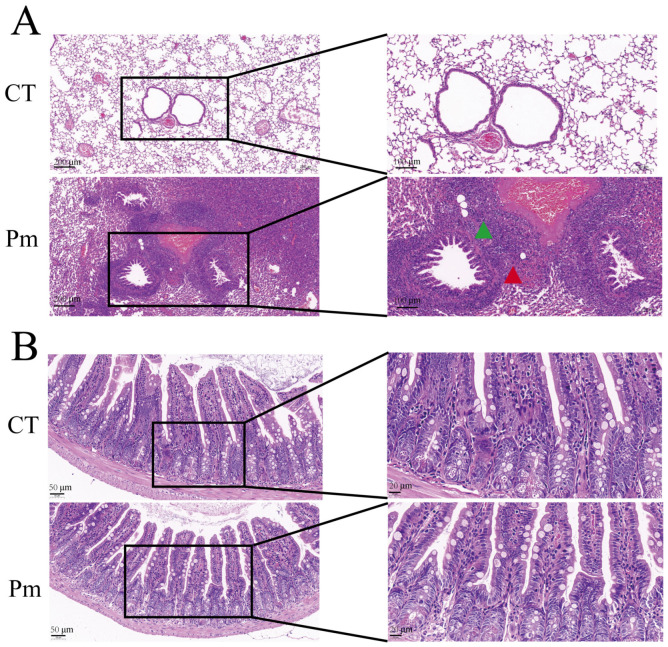
Histopathological examination of lung (**A**) and ileum (**B**) tissues stained with hematoxylin and eosin (H&E) (*n* = 6 per group). CT: control group; Pm: *NQ01*-infected group. Green triangles indicate areas of inflammatory cell infiltration, while red triangles denote bacterial foci.

At 24 hpi with NQ01, all mice in the Pm group exhibited clinical signs of HS to varying degrees, including dyspnea, ocular swelling, and subcutaneous edema. All mice were humanely euthanized at 36 hours post-infection (hpi) for the assessment of structural changes in internal organs. After infection, the lungs of the Pm mice demonstrated acute bronchiolitis-like pneumonia-related lesions. Specifically, there were many inflammatory cells infiltrating the bronchioles and the accompanying pulmonary veins, and necrotic tissues and bacterial foci could be observed in between. Similar to the control group, the ileal structure of the infected mice remained intact with clear boundaries. Specifically, the mucosal layer exhibited well-defined villi, and the intestinal epithelial cells were arranged in an orderly pattern with normal morphology, interspersed with scattered goblet cells. Paneth cells within the intestinal crypts exhibited normal secretory activity. Central lacteals in the lamina propria contained a typical distribution of lymphocytes, with no evident increase in inflammatory cell infiltration. The submucosal fibrous connective tissue appeared normal, without inflammatory cell infiltration (Figure 1).

### 3.2. NQ01 Infection Alters the Profile of Gut Microbiota in Mice

**Figure 2:**
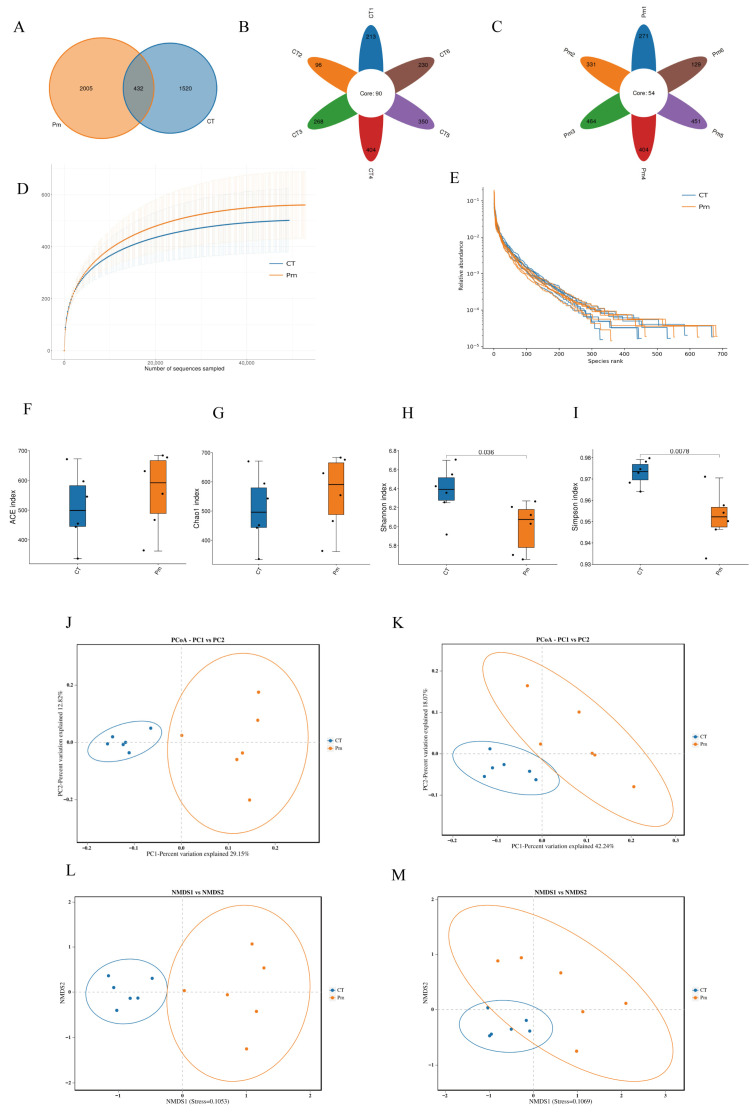
The effect of NQ01 on the composition and profile of the gut microbiota in mice. (**A**) The Venn diagram shows the quantity of the unique and shared ASVs in the CT and Pm groups. (**B**,**C**) The ASV floral diagram shows the quantity of ASVs in each group. (**D**) The rarefaction curve shows the features of each sample. (**E**) The alpha diversity index of the gut microbiota is visualized, respectively, i.e., ACE, Chao 1, Simpson, and Shannon. (**F**–**I**) Principal coordinates analysis (PCoA) was performed on the gut microbiota using unweight and weight Unifrac distance. **(J**–**M**) Non-metric multidimensional scaling (NMDS) analysis was performed to support the PCoA results. CT, the control group; Pm, the NQ01-infected group.

To assess the impact of *NQ01* infection on gut microbiota composition, 16S rRNA gene sequencing was performed on cecal contents from mice in the CT (control) and Pm (*NQ01*-infected) groups. There were 694,878 non-chimeric reads (CT = 352,812; Pm = 342,066) obtained from the 952,789 raw reads. All V3/V4 regions were clustered into 3957 ASVs. The Venn diagram described 432 ASVs shared between the two experimental groups, with 1520 and 2005 ASVs being exclusively identified in the CT group and Pm group, respectively (Figure 2A). The CT and Pm groups exhibited 90 and 54 core ASVs, respectively (Figure 2B,C). The rarefaction curve and rank-abundance curve reached plateaus, indicating that the sequencing depth was sufficient to capture the full bacterial species present in the samples (Figure 2D,E). Alpha index (ACE, Chao 1, Shannon, and Simpson) serves as a comprehensive metric for assessing species abundance and diversity. In this study, the ACE and Chao1 indices showed no significant differences (*p* > 0.05), whereas the Shannon index (*p* < 0.05) and Simpson index (*p* < 0.01) exhibited statistically significant variations. These results indicate that, although the richness of the gut microbiota remained largely unaffected following *NQ01* infection, its diversity was significantly decreased (Figure 2F–I). To further investigate structural differences in the gut microbiota between the CT and Pm groups, β-diversity was assessed using both weighted and unweighted UniFrac distances. Principal coordinate analysis (PCoA) based on these metrics revealed clear separation between the two groups (Figure 2J,K). Though the points in the CT group overlapped more than those in the Pm group, the NMDS plot also showed a relatively distinct clustering pattern (Figure 2L,M). As the beta analysis showed, NQ01 infection significantly altered the composition and profile of the gut microbiota in mice.

### 3.3. NQ01 Reshapes the Composition and Abundance of Gut Microbiota in Mice

**Figure 3:**
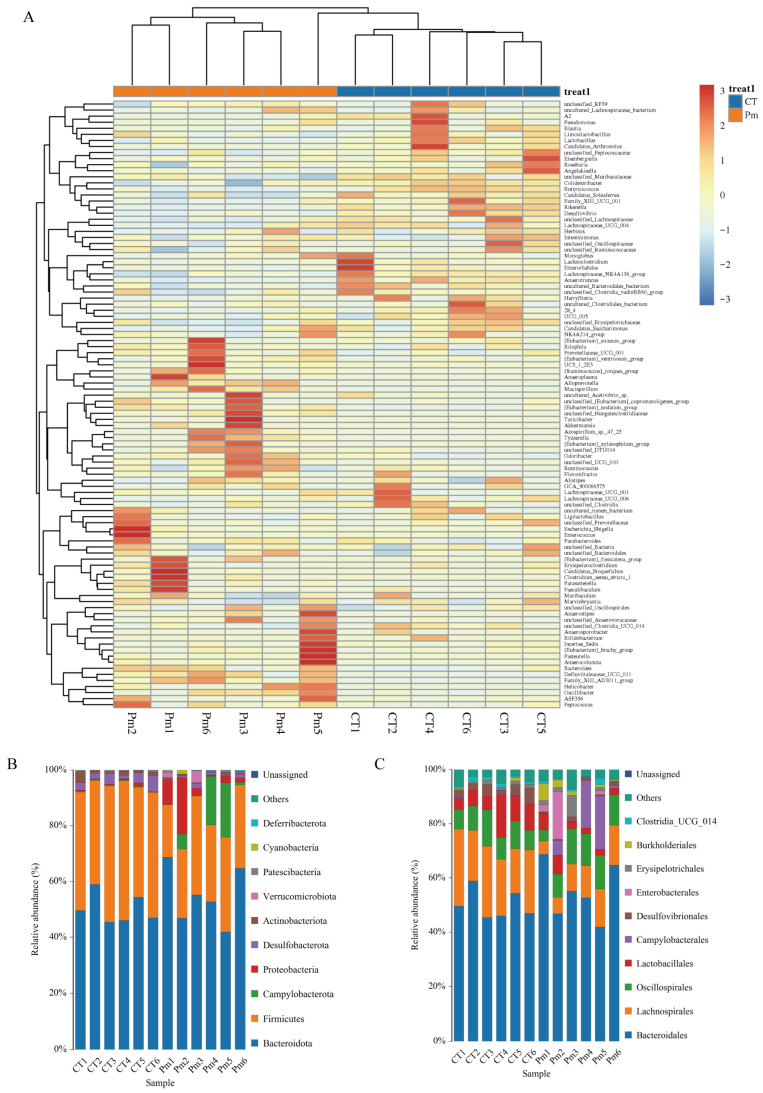
NQ01 infection changed the abundance and composition of the gut microbiota. (**A**) The heatmap depicted the dominant bacterial communities at the genus level. (**B**,**C**) The relative proportion and abundance of the dominant bacterial taxa at the phylum (**B**) and genus level (**C**), respectively.

To assess changes in gut microbiota composition, we examined the relative abundance of dominant taxa at multiple taxonomic levels. Across the 12 samples, 17 bacterial phyla and 212 genera were identified, with individual samples containing between 8 and 13 phyla and 88 and 120 genera (Figure 3A). At the phylum level, Bacteroidota (CT: 50.26%, Pm: 55.38%) and Firmicutes (CT: 43.82%, Pm: 28.47%) were the dominant composition, accounting for over 83% of the overall bacterial composition. Campylobacterota (CT: 2.83%, Pm: 7.00%), Proteobacteria (CT: 3.80%, Pm: 5.95%), and Actinobacteriota (CT: 1.53%, Pm: 3.96%) were identified as the other major composition. At the genus level, unclassified *Muribaculaceae* (CT: 27.73%, Pm: 20.00%), *Lachnospiraceae NK4A136* group (CT: 9.08%, Pm: 3.47%), *Alloprevotella* (CT: 0.57%, Pm: 10.15%), *Alistipes* (CT: 5.06%, Pm: 5.25%), *Muribaculum* (CT: 5.17%, Pm: 4.81%), *Lactobacillus* (CT: 5.89%, Pm: 1.55%), *Helicobacter* (CT: 0.28%, Pm: 7.00%), *28_4* (CT: 4.80%, Pm: 1.24%), uncultured *Bacteroidales* bacterium (CT: 3.71%, Pm: 10.15%), and *Prevotellaceae UCG 001* (CT: 0.57%, Pm: 10.15%) were enriched in the groups (Figure 3B,C).

**Figure 4:**
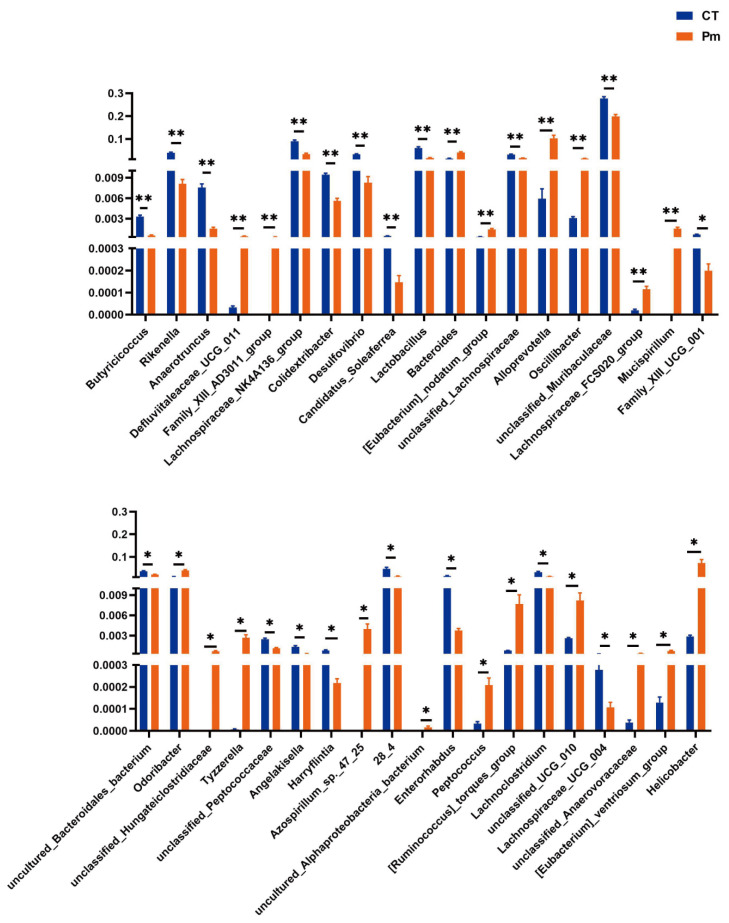
Metastats analysis was used to identify the differential bacterial taxa between two groups at the genus level. Rows represent individual bacterial taxa, and columns display the calculated mean value for each group. CT, the control group; Pm: the NQ01-infected group. Data are represented as means ± SD. “*” shows the significance level. * *p* < 0.05, ** *p* < 0.01.

To further identify differential bacterial biomarkers, Metastats analysis was performed to detect gut microbial taxa with significantly altered abundance. At the phylum level, *Firmicutes*, *Desulfobacterota*, *Deferribacterota*, *Actinobacteriota*, *Campylobacterota*, and *Proteobacteria* exhibited significant differences between the CT and Pm groups. At the genus level, 38 taxa were found to differ significantly in relative abundance between the two groups (Figure 4). Among them, 19 genera were decreased after NQ01 infection, while the relative abundance of the other 19 genera were relatively increased (*p* < 0.05). Additionally, Family *XIII AD3011* group, *Mucispirillum,* unclassified *Hungateiclostridiaceae*, *Azospirillum* sp. 47_25, and uncultured *Alphaproteobacteria* bacterium were undetectable before NQ01 infection. Although the genus *Pasteurella* was detected in the heatmap, no significant difference in its abundance was observed between the groups. These findings indicate that *NQ01* infection reshaped the gut microbiota structure in mice, leading to significant alterations in microbial composition and relative abundance.

### 3.4. NQ01 Infection Disrupted the Gut Microbiota Metabolism in Mice

**Figure 5:**
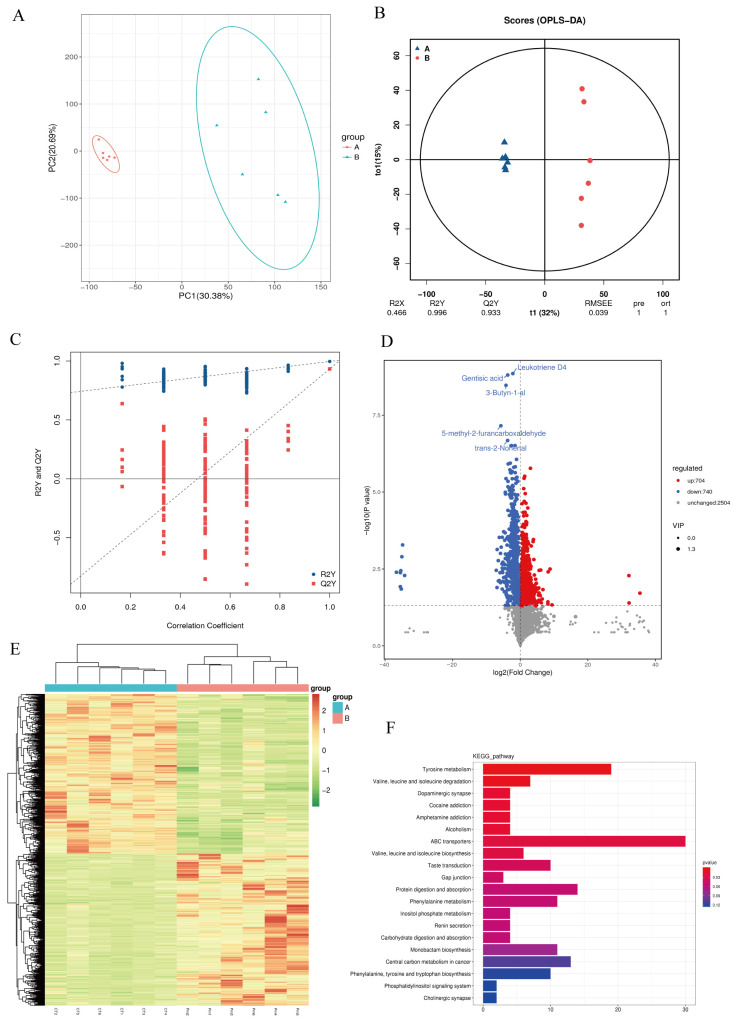
NQ01 infection changed the gut metabolic profiles and relative pathways in mice. (**A**) The principal component analysis plot. (**B**,**C**) The orthogonal projections to latent structures discriminant analysis (OPLS-DA) (**B**) and the permutation test (**C**). (**D**) The volcano map of the differential metabolites. (**E**) The heatmap of the differential metabolites. The horizontal axis represents samples, and the vertical axis represents the differential metabolites. (**F**) The pathways enriched by the differential metabolites in KEGG.

The gut microbiota and their metabolites are key regulators of intestinal homeostasis. To explore the metabolic disturbances caused by *NQ01* infection, we conducted untargeted metabolomic profiling of cecal contents, identifying a total of 3948 metabolites. Principal component analysis (PCA) revealed a clear separation between the CT and Pm groups, suggesting significant infection-associated metabolic alterations (Figure 5A). The OPLS-DA scatterplot also indicated the separation between two groups. The permutation test for OPLS-DA showed a positive slope for the regression line of the Q2Y, and the original Q2Y point on the right was higher than Q2Y values on the left, which strongly validates the reliability of the OPLS-DA model (Figure 5B,C). These findings suggest that the gut metabolism was disrupted by NQ01 infection. Moreover, the variable importance in the projection (VIP) was calculated based on the OPLS-DA model, which was used to screen the differential metabolites. Under the conditions of VIP > 1.0 and *p* < 0.05, a total of 1444 differential metabolites were identified (Figure 5D).

**Figure 6:**
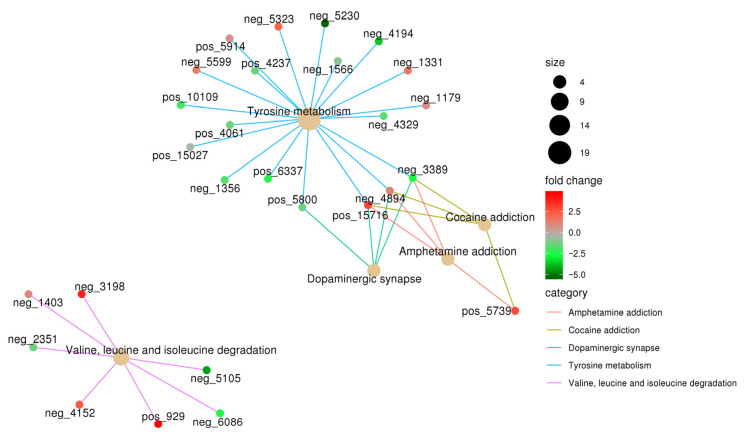
The network diagram displays the relationships between differential metabolites and pathways. neg: in negative-ion mode; pos: in positive-ion mode.

All differential metabolites were visualized using a clustered heatmap (Figure 5E). Among them, 704 were upregulated and 740 were downregulated after NQ01 infection. The differential metabolites were subjected to pathway analysis, and the representative enriched pathways are shown in Figure 5F. The top five enriched pathways were tyrosine metabolism; valine, leucine, and isoleucine degradation; dopaminergic synapse; cocaine addiction; and amphetamine addiction. A total of 27 metabolites involved in these pathways were identified, including 4-hydroxyphenylacetaldehyde, 5,6-dihydroxyindole, 3,4-dihydroxymandelate, N-methyltyramine, 4-(L-alanin-3-yl)-2-hydroxy-cis,cis-muconate 6-semialdehyde, tyrosol, 4-hydroxyphenylacetylglutamic acid, *p*-hydroxyphenylacetylglycine, tyramine, L-normetanephrine, 4-hydroxycinnamic acid, gentisic acid, L-dopachrome, maleic acid, L-adrenaline, 3,4-dihydroxy-L-phenylalanine (L-DOPA), dopamine, L-tyrosine, 3-methoxytyramine, L-glutamic acid, 3-methyl-2-oxobutanoic acid, (R)-3-amino-2-methylpropanoate, L-isoleucine, (S)-3-methyl-2-oxopentanoic acid, 2-methyl-3-oxopropanoate, L-leucine, and L-valine (Table 1). The associations between these biomarkers and metabolic pathways are visualized using an enrichment network diagram (Figure 6).

### 3.5. The Correlation Analysis for Differential Microbiota and Metabolites

**Figure 7:**
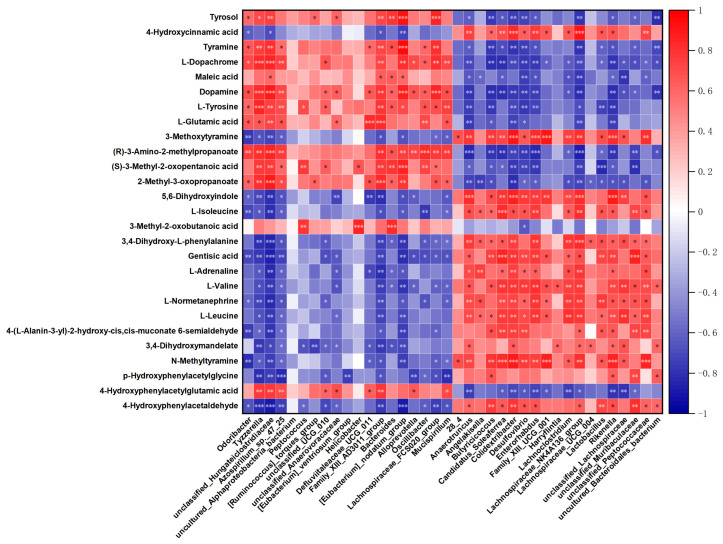
The relationship between the differential microbial genera and the differential metabolites. The horizontal axis represents the differential microbial genera, and the vertical axis represents the differential metabolites. “*” shows the significance level. * *p* < 0.05, ** *p* < 0.01, *** *p* < 0.001.

To further investigate the relationship between gut microbiota and metabolites, Spearman’s correlation analysis was performed to construct an interaction heatmap (Figure 7). As shown in the heatmap, *Peptococcus*, Family *XIII AD3011* group, *Eubacterium nodatum* group, and *Mucispirillum* were positively correlated with increased levels of L-tyrosine, whereas L-tyrosine was negatively correlated with *Butyricicoccus*, *Colidextribacter*, *Desulfovibrio*, *Enterorhabdus*, *Lachnospiraceae NK4A136* group, and *Rikenella*. While dopamine exhibited a correlation profile similar to that of L-tyrosine, it displayed distinct associations at the genus level—showing no significant correlation with *Peptococcus* and a negative association with unclassified *Anaerovoracaceae*. In contrast, 3,4-dihydroxy-L-phenylalanine (L-DOPA) exhibited an opposite trend: Family *XIII AD3011* group, *Eubacterium nodatum* group, and *Mucispirillum*, which were increased, showed negative correlations with the downregulated L-DOPA, while *Butyricicoccus*, *Colidextribacter*, *Enterorhabdus*, *Lachnospiraceae NK4A136* group, and *Lactobacillus* were positively correlated with it. Furthermore, the three branched-chain amino acids (L-valine, L-leucine, and L-isoleucine) displayed consistent correlation patterns with the differentially abundant microbial taxa. As three amino acids had downregulation, *Butyricicoccus*, Candidatus *Soleaferrea*, *Colidextribacter*, *Desulfovibrio, Enterorhabdus, Lachnospiraceae NK4A136* group, and *Rikenella* had a significant positive correlation. Though 3-methyl-2-oxobutanoic acid only had a significant negative correlation to *Desulfovibrio*, it was positively related to *Peptococcus*, *Helicobacter,* and *Bacteroides.* Additionally, upregulated (R)-3-amino-2-methylpropanoate was positively correlated to *Family XIII AD3011* group, *Eubacterium nodatum* group, *Lachnospiraceae FCS020* group, and *Mucispirillum*. It is also demonstrated that (R)-3-amino-2-methylpropanoate was negatively correlated to *Butyricicoccus*, Candidatus *Soleaferrea*, *Colidextribacter*, *Desulfovibrio, Enterorhabdus, Lachnospiraceae NK4A136* group, and *Rikenella*.

## 4. Discussion

Microbial colonization is a major driving factor, and the host phenotype is also profoundly influenced by the composition and abundance of the microbiota, as well as the diverse range of metabolites they produce [26]. In this study, *Pasteurella multocida* was administered via the respiratory tract to mimic the natural route of infection. Histopathological analysis revealed that *NQ01* infection induced significant lung injury in mice, consistent with our previous findings [25]. However, NQ01 infection caused no substantial ileal damage. Despite this, the acute respiratory infection induced abrupt gut microbiota alterations, which appear to have disrupted host homeostasis. Following *P. multocida* infection, the intestinal stress was generated by the gut microbiota disruption. Alpha diversity analysis demonstrated that there were no significant changes in the ACE and Chao 1 indices, suggesting that the total number of detected species was similar in both groups. However, the decline in Shannon and Simpson indices indicated a profound change in the community structure after infection. Although the number of unique ASVs in the Pm group was higher than that in the control group, the Shannon index and Simpson index are sensitive to both species richness and the uniformity of their distribution, and the marked decrease in evenness due to this population shift outweighed the contribution from the increased number of unique ASVs, resulting in an overall lower effective diversity. These results suggest that the increase in unique low-abundance ASVs in the Pm group may represent opportunistic or condition-specific taxa, which is consistent with the ecological scenario that pathogen challenge disrupts the stable microbiota, leading to a loss of dominant commensals and the emergence of numerous rare, low-abundance opportunistic taxa.

Consistent with previous reports, the gut microbiota at the phylum level was predominantly composed of *Bacteroidota* and *Firmicutes*, whereas greater variability was observed at the genus level [27]. In our study, *Mucispirillum* and *Defluviitaleaceae UCG 011* were upregulated, and this trend also associated with promoting the progression of Crohn’s disease, regarded as a harmful bacterial genus [28,29]. We also observed an increase in the relative abundance of *Peptococcus*, a genus associated with lung diseases. *Peptococcus* was enriched in the intestines of non-small-cell lung cancer patients and was considered as a potential biomarker [30]. Similarly, bacterial genera such as unclassified *Anaerovoracaceae* and the *Eubacterium nodatum* group are positively correlated with the sub-health or disease states, and also showed increased relative abundance [31,32,33,34]. In contrast, the relative abundance of several beneficial bacteria decreased, such as *Colidextribacter*, *Butyricicoccus*, *Enterorhabdus,* Family *XIII UCG 001*, *Harryflintia*, and the *Lachnospiraceae NK4A136* group, which were associated with SCFAs synthesis [35,36,37,38,39,40]. These results indicate that *P. multocida* infection disrupts the gut microbiota, characterized by an increase in potentially pathogenic bacteria and a reduction in beneficial microbes capable of synthesizing short-chain fatty acids (SCFAs), which may, in turn, contribute to the progression of hemorrhagic septicemia (HS). Intestinal bacteria can secrete a diverse array of metabolites with distinct biological functions, which serve as crucial signaling molecules in the communication between the gut microbiota and host cells [41]. These metabolites can further enter the portal vein and systemic circulation to act on distant organs such as the liver, kidneys, and brain, thereby orchestrating systemic host homeostasis and potentially eliciting whole-body physiological effects [42]. As we observed, the abundance of *P. multocida* in the gut had no significant change, and seemingly no translocation to the intestine; therefore, there was another way to affect the intestinal homeostasis. In our study, the differential metabolites were enriched in tyrosine metabolism, dopaminergic synapse, cocaine addiction, and amphetamine addiction, which share the same three metabolites’ nodes: L-tyrosine, dopamine, and L-DOPA. Phenylalanine is the precursor for the synthesis of tyrosine, which is also decreased by *P. multocida* infection (log_2_FC = 0.722, *p* < 0.001). L-tyrosine serves as the direct precursor for L-DOPA synthesis, a process in which tetrahydrobiopterin acts as an essential cofactor [43]. Subsequently, L-DOPA is decarboxylated by the enzyme dopa decarboxylase to form dopamine. In this study, L-DOPA was downregulated while L-tyrosine and dopamine were upregulated, along with the reduced tyrosine hydroxylase activity, and it is also confirmed from previous study [44]. Tyrosine is converted into tyramine under the action of decarboxylase and then further oxidized and metabolized into tyrosol, in which both of these reaction products are upregulated. These dysregulated metabolites collectively point to a disruption in the tyrosine metabolism pathway, which may represent a key mechanism through which HS induces systemic symptoms. Tyrosine itself serves as the pivotal metabolite within this pathway. Glutamate is widely recognized as an excitatory transmitter. When encountering injury or stress, a high concentration of glutamate is released from the inside of the cell [45]. Here, we observe an abnormal enrichment of L-glutamic acid, which might be caused by the remodeling of the gut microbiota. In addition, the pathway of valine, leucine, and isoleucine degradation was also enriched. L-valine, L-leucine, and L-isoleucine all belong to branched-chain amino acids (BCAAs), which are important substrates for the biosynthesis of branched-chain fatty acids (BCFAs). Followed by *P. multocida* infection, L-valine, L-leucine, and L-isoleucine were downregulated, while the necessary precursors for synthesizing SCFAs were upregulated, indicating that the biosynthesis of SCFAs and BCFAs were blocked, which may impact the health of the host [46,47].

The correlation analysis of the differential microbiota and metabolites further elucidated the synergistic or antagonistic regulatory relationships. In the intestines of MD4 mice without specific antibodies, L-tyrosine was efficiently converted by the gut microbiota into p-cresol sulfate, then it provided protection against allergic asthma [48]. The above research provides direct evidence of the involvement of the gut microbiota in tyrosine metabolism. Another study showed that *Peptococcus* regulates smoking addiction (which can be regarded as a model of long-term exposure of the respiratory tract to cancer-causing agents) through tyramine and tryptophan metabolism [49]. Our results are consistent with this trend, showing a positive correlation between *Peptococcus* and tyrosine levels. Moreover, as previously reported, the Family *XIII AD3011* group had a negative correlation with phenylalanine level in a phenylketonuria disease model, which is a similar observation to our study; meanwhile, it had a positive correlation with L-tyrosine [50].

To our knowledge, this study is the first to report that HS-associated *P. multocida* infection induces gut microbiota remodeling and disrupts intestinal amino acid metabolism, identifying tyrosine metabolism as the centrally affected pathway. To better understand the conditional pathogenicity of *P. multocida*, it is necessary to identify the symbiotic bacteria and establish the correlation with the lung microbiota and metabolism. However, these insights underscore the importance of the gut–lung axis in acute bacterial infections. Our study provides the first clear evidence that localized *P. multocida* pneumonia can swiftly perturb distal gut microbial communities and metabolic profiles without direct gastrointestinal infection, likely through immune-mediated or stress-induced pathways. This systemic ripple effect of a lung infection emphasizes that HS is not merely a respiratory disease but a condition with body-wide implications. The pronounced disruption of tyrosine metabolism, in particular, suggests a potential mechanistic link between gut dysbiosis and the systemic symptoms observed in HS, as alterations in neurotransmitter precursors (like dopamine) could contribute to the illness’s severity. Understanding these cross-talk mechanisms will be crucial moving forward. Future research should focus on unraveling the molecular and immunological signals that connect respiratory infections to gut microbiota changes. For instance, identifying which host inflammatory mediators or microbial components drive the gut dysbiosis will help clarify how lung infections exert control over intestinal homeostasis. It will also be valuable to investigate the reciprocal side of this axis: how changes in the gut microbiota might feed back to influence lung immunity and infection outcomes. Additionally, correlating the gut microbial alterations with any shifts in the lung’s own microbiome or the host’s metabolic responses could deepen our understanding of HS’s systemic nature. Ultimately, these findings open up new avenues for therapeutic intervention: by targeting the microbiome or its metabolites, we might mitigate the broader impacts of hemorrhagic septicemia. For example, strategies to preserve or restore beneficial SCFA-producing bacteria in the gut could potentially buffer the host against the full brunt of the infection. In summary, our study highlights a complex, integrated host response in which a respiratory *P. multocida* infection disrupts gut microbial and metabolic equilibrium, and it lays the groundwork for exploring microbiota-centered approaches to improve outcomes in HS and other acute infectious diseases.

## 5. Conclusions

Infection with *P. multocida* NQ01 (serotype B:2), a key agent of hemorrhagic septicemia (HS), profoundly disrupts the host’s gut microbiota and metabolism. Despite sparing the intestinal lining from visible damage, the infection led to reduced microbial diversity and a shift toward proinflammatory and opportunistic taxa, alongside depletion of beneficial SCFA-producing bacteria. Metabolomic analysis revealed significant dysregulation of amino acid pathways, particularly tyrosine and branched-chain amino acids, impairing SCFA biosynthesis and intestinal nutrient balance. These findings uncover a previously underrecognized gut-mediated component in HS pathogenesis, linking microbial and metabolic imbalances to disease progression. Collectively, our findings reveal that *P. multocida* infection significantly remodels the gut microbiota and disrupts metabolic equilibrium, underscoring an underrecognized intestinal dimension in the pathogenesis of HS. These insights provide a foundation for future investigations into gut–lung and gut–systemic interactions in HS and highlight the therapeutic potential of targeting microbial and metabolic pathways to mitigate disease severity. Additionally, this study used a single mouse model, one *P. multocida* strain, and an acute time window, so the findings may not fully represent the broader, long-term disease dynamics across hosts and strains.

## Figures and Tables

**Table 1:** Differential metabolites in the top 5 enriched pathways.

Number	Name	log_2_FC	*p* Value	VIP	Trend
pos6337	4-Hydroxyphenylacetaldehyde	−2.4	0.0001	1.67	down
neg1356	5,6-Dihydroxyindole	−1.71	4.13 × 10^−5^	1.64	down
pos15027	3,4-Dihydroxymandelate	−0.5	0.0210	1.16	down
pos4061	N-Methyltyramine	−1.31	0.0024	1.43	down
pos10109	4-(L-Alanin-3-yl)-2-hydroxy-cis,cis-muconate 6-semialdehyde	−1.96	0.0065	1.36	down
neg5599	Tyrosol	1.31	0.0466	1.09	up
pos5914	4-Hydroxyphenylacetylglutamic acid	0.72	0.0003	1.56	up
pos4237	p-Hydroxyphenylacetylglycine	−1.35	0.0107	1.24	down
neg5323	Tyramine	2.06	0.0211	1.25	up
neg5230	L-Normetanephrine	−5.31	0.0030	1.53	down
neg1566	4-Hydroxycinnamic acid	−0.92	0.0127	1.29	down
neg4194	Gentisic acid	−3.7	1.55 × 10^−9^	1.76	down
neg1331	L-Dopachrome	1.49	0.0024	1.54	up
neg1179	Maleic acid	0.81	0.0187	1.26	up
neg4329	L-Adrenaline	−1.63	0.0038	1.42	down
neg3389	3,4-Dihydroxy-L-phenylalanine	−2.56	1.28 × 10^−5^	1.69	down
neg4894	Dopamine	1.09	0.0112	1.39	up
pos15716	L-Tyrosine	2.99	0.0105	1.33	up
pos5800	3-Methoxytyramine	−1.3	0.0054	1.36	down
pos5739	L-Glutamic acid	2.77	0.0103	1.24	up
neg3198	3-Methyl-2-oxobutanoic acid	3.77	0.0421	1.15	up
neg1403	(R)-3-Amino-2-methylpropanoate	0.92	0.0003	1.53	up
neg2351	L-Isoleucine	−1.38	0.0013	1.47	down
neg4152	(S)-3-Methyl-2-oxopentanoic acid	2.33	0.0094	1.39	up
pos929	2-Methyl-3-oxopropanoate	4.63	0.0096	1.45	up
neg6086	L-Leucine	−2.5	0.0022	1.5	down
neg5105	L-Valine	−4.37	0.0011	1.6	down

## Data Availability

Raw sequencing data generated in this study are publicly available in the NCBI Sequence Read Archive (SRA) under accession number PRJNA1390072. The original contributions presented in this study are included in the article. Further inquiries can be directed to the corresponding author.

## References

[1] Peng Z., Wang X., Zhou R., Chen H., Wilson B.A., Wu B. (2019). *Pasteurella multocida*: Genotypes and Genomics. Microbiol. Mol. Biol. Rev..

[2] Carter G.R. (1955). Studies on *Pasteurella multocida*. I. A hemagglutination test for the identification of serological types. Am. J. Vet. Res..

[3] Heddleston K.L., Gallagher J.E., Rebers P.A. (1972). Fowl cholera: Gel diffusion precipitin test for serotyping *Pasteurella multocida* from avian species. Avian Dis..

[4] Townsend K.M., Boyce J.D., Chung J.Y., Frost A.J., Adler B. (2001). Genetic organization of *Pasteurella multocida* cap Loci and development of a multiplex capsular PCR typing system. J. Clin. Microbiol..

[5] Dabo S.M., Taylor J.D., Confer A.W. (2007). *Pasteurella multocida* and bovine respiratory disease. Anim. Health Res. Rev..

[6] Chanda M.M., Purse B.V., Hemadri D., Patil S.S., Yogisharadhya R., Prajapati A., Shivachandra S.B. (2024). Spatial and temporal analysis of haemorrhagic septicaemia outbreaks in India over three decades (1987–2016). Sci. Rep..

[7] Almoheer R., Abd Wahid M.E., Zakaria H.A., Jonet M.A.B., Al-Shaibani M.M., Al-Gheethi A., Addis S.N.K. (2022). Spatial, Temporal, and Demographic Patterns in the Prevalence of Hemorrhagic Septicemia in 41 Countries in 2005–2019: A Systematic Analysis with Special Focus on the Potential Development of a New-Generation Vaccine. Vaccines.

[8] Lestari T.D., Khairullah A.R., Damayanti R., Mulyati S., Rimayanti R., Hernawati T., Utama S., Kusuma Wardhani B.W., Wibowo S., Ariani Kurniasih D.A. (2025). Hemorrhagic septicemia: A major threat to livestock health. Open Vet. J..

[9] Rhoades K.R., Heddleston K.L., Rebers P.A. (1967). Experimental hemorrhagic septicemia: Gross and microscopic lesions resulting from acute infections and from endotoxin administration. Can. J. Comp. Med. Vet. Sci..

[10] Wilkie I.W., Harper M., Boyce J.D., Adler B. (2012). *Pasteurella multocida*: Diseases and pathogenesis. Curr. Top. Microbiol. Immunol..

[11] Barcik W., Boutin R.C.T., Sokolowska M., Finlay B.B. (2020). The Role of Lung and Gut Microbiota in the Pathology of Asthma. Immunity.

[12] Budden K.F., Gellatly S.L., Wood D.L., Cooper M.A., Morrison M., Hugenholtz P., Hansbro P.M. (2017). Emerging pathogenic links between microbiota and the gut-lung axis. Nat. Rev. Microbiol..

[13] Ichinohe T., Pang I.K., Kumamoto Y., Peaper D.R., Ho J.H., Murray T.S., Iwasaki A. (2011). Microbiota regulates immune defense against respiratory tract influenza A virus infection. Proc. Natl. Acad. Sci. USA.

[14] Brown R.L., Sequeira R.P., Clarke T.B. (2017). The microbiota protects against respiratory infection via GM-CSF signaling. Nat. Commun..

[15] Fox A.C., McConnell K.W., Yoseph B.P., Breed E., Liang Z., Clark A.T., O’Donnell D., Zee-Cheng B., Jung E., Dominguez J.A. (2012). The endogenous bacteria alter gut epithelial apoptosis and decrease mortality following *Pseudomonas aeruginosa* pneumonia. Shock.

[16] Wang L., Cai Y., Garssen J., Henricks P.A.J., Folkerts G., Braber S. (2023). The Bidirectional Gut-Lung Axis in Chronic Obstructive Pulmonary Disease. Am. J. Respir. Crit. Care Med..

[17] Song X.L., Liang J., Lin S.Z., Xie Y.W., Ke C.H., Ao D., Lu J., Chen X.M., He Y.Z., Liu X.H. (2024). Gut-lung axis and asthma: A historical review on mechanism and future perspective. Clin. Transl. Allergy.

[18] Narayana J.K., Aliberti S., Mac Aogáin M., Jaggi T.K., Ali N., Ivan F.X., Cheng H.S., Yip Y.S., Vos M.I.G., Low Z.S. (2023). Microbial Dysregulation of the Gut-Lung Axis in Bronchiectasis. Am. J. Respir. Crit. Care Med..

[19] Layeghifard M., Li H., Wang P.W., Donaldson S.L., Coburn B., Clark S.T., Caballero J.D., Zhang Y., Tullis D.E., Yau Y.C.W. (2019). Microbiome networks and change-point analysis reveal key community changes associated with cystic fibrosis pulmonary exacerbations. npj Biofilms Microbiomes.

[20] Dickson R.P., Singer B.H., Newstead M.W., Falkowski N.R., Erb-Downward J.R., Standiford T.J., Huffnagle G.B. (2016). Enrichment of the lung microbiome with gut bacteria in sepsis and the acute respiratory distress syndrome. Nat. Microbiol..

[21] Wang Y., Malmuthuge N., Yao J., Guan L.L. (2025). Revisiting cattle respiratory health: Key roles of the gut-lung axis in the dynamics of respiratory tract pathobiome. Microbiol. Mol. Biol. Rev..

[22] Trompette A., Gollwitzer E.S., Yadava K., Sichelstiel A.K., Sprenger N., Ngom-Bru C., Blanchard C., Junt T., Nicod L.P., Harris N.L. (2014). Gut microbiota metabolism of dietary fiber influences allergic airway disease and hematopoiesis. Nat. Med..

[23] Bogard G., Makki K., Brito-Rodrigues P., Tan J., Molendi-Coste O., Barthelemy J., Descat A., Bouilloux F., Lecoeur C., Grangette C. (2025). Impact of aging on gut-lung-adipose tissue interactions and lipid metabolism during influenza infection in mice. Sci. Rep..

[24] Ho S.Y., Lee C.Y., Chou H.C., Huang L.T., Chen C.M. (2025). Maternal Aspartame Exposure Induces Neonatal Pulmonary Metabolic Dysregulation and Redox Imbalance: A Multiomics Investigation of Gut Microbiota-Host Interactions. J. Agric. Food Chem..

[25] Li K., Yuan H., Jin C., Rahim M.F., Luosong X., An T., Li J. (2025). Characterization and Genomic Analysis of *Pasteurella multocida* NQ01 Isolated from Yak in China. Animals.

[26] Jandhyala S.M., Talukdar R., Subramanyam C., Vuyyuru H., Sasikala M., Nageshwar Reddy D. (2015). Role of the normal gut microbiota. World J. Gastroenterol..

[27] Arumugam M., Raes J., Pelletier E., Le Paslier D., Yamada T., Mende D.R., Fernandes G.R., Tap J., Bruls T., Batto J.-M. (2011). Enterotypes of the human gut microbiome. Nature.

[28] Caruso R., Mathes T., Martens E.C., Kamada N., Nusrat A., Inohara N., Núñez G. (2019). A specific gene-microbe interaction drives the development of Crohn’s disease-like colitis in mice. Sci. Immunol..

[29] Huang Y.L., Zheng J.M., Shi Z.Y., Chen H.H., Wang X.T., Kong F.B. (2024). Inflammatory proteins may mediate the causal relationship between gut microbiota and inflammatory bowel disease: A mediation and multivariable Mendelian randomization study. Medicine.

[30] Chen J., Yu X., Wu X., Chai K., Wang S. (2024). Causal relationships between gut microbiota, immune cell, and Non-small cell lung cancer: A two-step, two-sample Mendelian randomization study. J. Cancer.

[31] Khurshid H., Jamshaid M.B., Salahuudin Z., Sibtain K., Fayyaz I., Ameer A., Kerbiriou C., McKirdy S., Malik S.N., Gerasimidis K. (2025). Gut microbial ecology and function of a Pakistani cohort with Iron deficiency Anemia. Sci. Rep..

[32] Liu Y., Luo R., Sun Z., Zhang Y., Guo Y., Chen Y., Li L., Yue Z. (2025). Synergistic Toxicity of Combined Exposure to Acrylamide and Polystyrene Nanoplastics on the Gut-Liver Axis in Mice. Biology.

[33] Unrug-Bielawska K., Sandowska-Markiewicz Z., Piątkowska M., Czarnowski P., Goryca K., Zeber-Lubecka N., Dąbrowska M., Kaniuga E., Cybulska-Lubak M., Bałabas A. (2025). Comparative Analysis of Gut Microbiota Responses to New SN-38 Derivatives, Irinotecan, and FOLFOX in Mice Bearing Colorectal Cancer Patient-Derived Xenografts. Cancers.

[34] Zhang J.S., Li S., Cheng X., Tan X.C., Huang Y.L., Dong H.J., Xue R., Zhang Y., Li J.C., Feng X.X. (2024). Far-Infrared Therapy Based on Graphene Ameliorates High-Fat Diet-Induced Anxiety-Like Behavior in Obese Mice via Alleviating Intestinal Barrier Damage and Neuroinflammation. Neurochem. Res..

[35] Liu X., Zhang Y., Li W., Zhang B., Yin J., Liuqi S., Wang J., Peng B., Wang S. (2022). Fucoidan Ameliorated Dextran Sulfate Sodium-Induced Ulcerative Colitis by Modulating Gut Microbiota and Bile Acid Metabolism. J. Agric. Food Chem..

[36] Zhang P., Jiang G., Wang Y., Yan E., He L., Guo J., Yin J., Zhang X. (2023). Maternal consumption of l-malic acid enriched diets improves antioxidant capacity and glucose metabolism in offspring by regulating the gut microbiota. Redox Biol..

[37] Deng Y., Zhou M., Wang J., Yao J., Yu J., Liu W., Wu L., Wang J., Gao R. (2021). Involvement of the microbiota-gut-brain axis in chronic restraint stress: Disturbances of the kynurenine metabolic pathway in both the gut and brain. Gut Microbes.

[38] Song W., Wang Y., Li G., Xue S., Zhang G., Dang Y., Wang H. (2023). Modulating the gut microbiota is involved in the effect of low-molecular-weight Glycyrrhiza polysaccharide on immune function. Gut Microbes.

[39] Tirelli E., Pucci M., Squillario M., Bignotti G., Messali S., Zini S., Bugatti M., Cadei M., Memo M., Caruso A. (2025). Effects of methylglyoxal on intestine and microbiome composition in aged mice. Food Chem. Toxicol..

[40] Zhong S., Yang Y.N., Huo J.X., Sun Y.Q., Zhao H., Dong X.T., Feng J.Y., Zhao J., Wu C.M., Li Y.G. (2025). Cyanidin-3-rutinoside from Mori Fructus ameliorates dyslipidemia via modulating gut microbiota and lipid metabolism pathway. J. Nutr. Biochem..

[41] Cai J., Rimal B., Jiang C., Chiang J.Y.L., Patterson A.D. (2022). Bile acid metabolism and signaling, the microbiota, and metabolic disease. Pharmacol. Ther..

[42] Xie C., Zhu X., Xu B., Niu Y., Zhang X., Ma L., Yan X. (2022). Integrated analysis of multi-tissues lipidome and gut microbiome reveals microbiota-induced shifts on lipid metabolism in pigs. Anim. Nutr..

[43] Daubner S.C., Le T., Wang S. (2011). Tyrosine hydroxylase and regulation of dopamine synthesis. Arch. Biochem. Biophys..

[44] Fahn S. (2008). The history of dopamine and levodopa in the treatment of Parkinson’s disease. Mov. Disord..

[45] Moroz L.L., Nikitin M.A., Poličar P.G., Kohn A.B., Romanova D.Y. (2021). Evolution of glutamatergic signaling and synapses. Neuropharmacology.

[46] Abdallah A., Elemba E., Zhong Q., Sun Z. (2020). Gastrointestinal Interaction between Dietary Amino Acids and Gut Microbiota: With Special Emphasis on Host Nutrition. Curr. Protein Pept. Sci..

[47] Ezzine C., Loison L., Montbrion N., Bôle-Feysot C., Déchelotte P., Coëffier M., Ribet D. (2022). Fatty acids produced by the gut microbiota dampen host inflammatory responses by modulating intestinal SUMOylation. Gut Microbes.

[48] Wypych T.P., Pattaroni C., Perdijk O., Yap C., Trompette A., Anderson D., Creek D.J., Harris N.L., Marsland B.J. (2021). Microbial metabolism of L-tyrosine protects against allergic airway inflammation. Nat. Immunol..

[49] Fan J., Zhou Y., Meng R., Tang J., Zhu J., Aldrich M.C., Cox N.J., Zhu Y., Li Y., Zhou D. (2023). Cross-talks between gut microbiota and tobacco smoking: A two-sample Mendelian randomization study. BMC Med..

[50] Liu Z., Ye M., Jin H., Chen W., Jieens H., Han R. (2025). Relationship Between Gut Microbiota and Phenylalanine Levels: A Mendelian Randomization Study. Microbiologyopen.

